# The use of dronedarone for recurrent ventricular tachycardia: a case report and review of the literature

**DOI:** 10.1186/s13104-016-2116-1

**Published:** 2016-07-27

**Authors:** Jacques Rizkallah, Vikas Kuriachan, L. Brent Mitchell

**Affiliations:** 1 Libin Cardiovascular Institute of Alberta, University of Calgary, Calgary, Canada; 2 C829 Foothills Medical Centre, 1403 29 Street NW, Calgary, AB T2N 2T9 Canada

**Keywords:** Dronedarone, Ventricular tachycardia, Arrhythmia, Drug-refractory, Adverse drug effect

## Abstract

**Background:**

Dronedarone is a benzofuran derivative resembling amiodarone that was intended to reduce the iodine-associated tissue deposition and organ toxicity seen with the latter. The utility of dronedarone for patients with ventricular arrhythmias has not been thoroughly evaluated. We present our experience with its use to treat refractory ventricular tachycardia storm and review the literature.

**Case presentation:**

An 85 year-old gentleman with multiple medical comorbidities including ischemic and non-ischemic cardiomyopathy with severe biventricular systolic dysfunction presented with ventricular tachycardia storm. Therapeutic options were limited given his frail medical status, failures of sotalol, mexilitine, and catheter ablation therapies along with drug-toxicities from amiodarone. Dronedarone was thus considered as off-label use following informed consent. The patient unfortunately developed fatal multisystem organ failure including acute severe hepatotoxicity from dronedarone.

**Conclusion:**

Novel therapies for drug-refractory ventricular arrhythmias are long overdue given the limitations of available pharmacologic and non-pharmacologic options. Off-label use of antiarrhythmic agents such as dronedarone is considered a treatment of last-resort in patients who otherwise have no therapeutic options. Given the paucity of reported cases regarding dronedarone for the treatment of ventricular tachyarrhythmias, no conclusive recommendations can be made at this time aside from words of caution. Despite the potential ventricular antiarrhythmic effects of dronedarone, careful patient evaluation is required to identify those at greatest risk of drug-related adverse events particularly in those patients with significant comorbidities such as advanced hepatic, renal, and cardiovascular disease.

## Background

Amiodarone is one the most effective pharmacologic agents to treat ventricular arrhythmias however its use is limited by a high incidence of drug-related adverse events [1]. Dronedarone is a benzofuran derivative resembling amiodarone with the iodine moiety replaced by a methane-sulfonyl group [2]. Its development was intended to reduce the iodine-associated tissue deposition and organ toxicity of amiodarone [2]. The utility of dronedarone for patients with ventricular arrhythmias has not been thoroughly evaluated [3]. We present our experience with its use in a patient with refractory ventricular tachycardia (VT) storm and review the literature to further guide its use in this setting.

### Case presentation

An 85 year-old Caucasian gentleman with hypertension, paroxysmal atrial fibrillation, hypothyroidism, stage 3 chronic kidney disease, mixed ischemic and non-ischemic cardiomyopathy, severe biventricular systolic dysfunction, secondary prevention cardiac resynchronization therapy device implant with defibrillator function, and frequent monomorphic VT presents with VT storm. Heart failure medical therapy was optimized and included beta-blockade. A prior trial of sotalol therapy provided no arrhythmia suppression. Amiodarone therapy provided some relief, particularly when augmented by mexilitine therapy, however drug-related lung and neurologic toxicities limited amiodarone use. Transvenous catheter VT ablation was attempted however the arrhythmia recurred a month later.

On presentation, the patient had recurrent VT at 215 beats per minute. 12-lead electrocardiography revealed a monomorphic wide complex tachycardia with left bundle branch block like morphology, inferior axis, and late precordial transition. His arrhythmia precipitated frequent appropriate implantable cardioverter defibrillator anti-tachycardia pacing and shocks therapies. There was no evidence of ongoing reversible myocardial ischemia or metabolic and electrolyte abnormalities.

Given his severe biventricular failure, prior sotalol and mexilitine failure, and drug-toxicities with amiodarone, antiarrhythmic options were limited. The patient was not a good candidate for a repeat ablation procedure or cardiac transplantation given his age and frail status. Having exhausted antiarrhythmic options, dronedarone therapy was considered for off-label use. After discussing potential benefits and risks regarding dronedarone use in the treatment of VT based on the limited data available in the scientific literature, the patient consented to an off-label trial of the agent at 400 mg twice daily. After only three doses, it was discontinued due to severe nausea and the development of multisystem organ failure including acute severe hepatotoxicity. This brief trial of dronedarone therapy had no appreciable effect of the patient’s VT burden. The duration of the VT episodes was brief and not felt to be the cause of the patient’s multi-organ failure that was temporally associated with the initiation of dronedarone therapy. Five days prior to dronedarone administration, the patient’s serum alanine transaminase was measured at 59 U/L (normal 11–63 U/L), serum creatinine at 135 umol/L (normal 50–120 umol/L), and estimated glomerular filtration rate at 41 mL/min/1.73 m^2^ (normal >80 ml/min/1.73 m^2^). Two days after dronedarone administration a marked decline in kidney and liver function was observed with a serum alanine transaminase level increase to 1045 U/L, serum creatinine level of 231 umol/L, and estimate glomerular filtration rate of 21 ml/min/1.73 m^2^. Dronedarone therapy was subsequently discontinued. Unfortunately the patient’s liver and renal failure continued to worsen despite drug discontinuation. He died eight days later from multi-organ system failure and metabolic shock.

## Discussion

Only three cases have been published thus far in the medical literature documenting the successful use of dronedarone for recurrent VT [4–6] and no cases have been published documenting its failure in this regard.

An 81 year-old male with non-ischemic cardiomyopathy and severe left ventricular systolic dysfunction was receiving amiodarone and a beta-blocker in addition to optimized heart failure medical therapy but continued to have recurrent episodes of VT [4]. The use of amiodarone therapy was limited by thyroid toxicity requiring its discontinuation. Sotalol and propafenone were trialed and discontinued secondary to intolerance and procainamide therapy was unsuccessful. The patient declined VT catheter ablation and dronedarone was subsequently offered as an option of last-resort with the clear understanding of its off-label use. In this patient, dronedarone therapy was associated with a significant reduction in arrhythmia burden.

The second case of successful dronedarone use for VT therapy in severe LV systolic dysfunction was in a 42 year-old patient with “end-stage” hypertrophic cardiomyopathy presenting with recurrent hemodynamically-stable, monomorphic VT [5]. He was started on intravenous amiodarone that resulted in arrhythmia suppression. Given the patient’s young age and concerns for long-term toxicity risks with amiodarone therapy, dronedarone 400 mg twice daily was initiated and was well tolerated. The patient remained arrhythmia free after 24 months of follow-up.

The third case of successful dronedarone use for the suppression of VT was in a 41 year old gentleman with non-ischemic dilated cardiomyopathy, mild LV systolic dysfunction, and frequent monomorphic non-sustained VT episodes [6]. The patient had delayed enhancement on cardiac MRI in the basal segment of the interventricular septum consistent with scar-based cardiomyopathy in the absence of coronary artery disease. He subsequently had an endocardial VT ablation attempt which was not successful and continued to have recurrent tachycardia despite trials of multiple antiarrhythmic agents including sotalol, propafenone, mexiletine, quinidine, amiodarone, and ranolazine. His amiodarone therapy was discontinued due to concerns for long-term toxicities. Off-label dronedarone therapy was initiated and resulted in a significant reduction in arrhythmia burden after a 3 months follow-up period.

In distinction to these cases, our patient did not tolerate dronedarone and died from severe drug-related hepatotoxicity and metabolic shock. Although its use for the treatment of VT is off-label, in this setting it was a treatment of last resort.

A review of the literature regarding dronedarone will assist in its appropriate clinical use. Dronedarone was well studied in atrial fibrillation (AF) and guidance regarding its use to treat ventricular arrhythmias can be extrapolated from these well-designed studies. The ATHENA-investigators randomized 4628 patients with paroxysmal or persistent AF or flutter who had no severe heart failure symptoms to dronedarone 400 mg twice daily or placebo but only an estimated 4 % of patients had severe LV systolic dysfunction [2]. In addition to a significant reduction in AF recurrence (hazard ratio of 0.63), dronedarone reduced hospitalizations due to cardiovascular events or death likely by reducing ventricular arrhythmias. Dronedarone has also been documented to have ventricular antiarrhythmic effects in animal models with ischemia-induced arrhythmias [7, 8]. The most significant clinical adverse events of dronedarone were bradycardia, QT-prolongation, nausea, diarrhea, abnormal liver-function tests, and increased serum creatinine.

Dronedarone should be avoided or used with extreme caution in the setting of severe left ventricular dysfunction. In a randomized double-blind trial of 637 hospitalized patients with symptomatic heart failure and severe left ventricular dysfunction therapy with dronedarone 400 mg twice daily increased early mortality secondary to progressive heart failure particularly in those with severe systolic dysfunction [9]. These observations suggest that dronedarone may directly or indirectly worsen left ventricular systolic function. Patients treated with dronedarone were also noted to have a 10–20 % increase in serum creatinine that is considered to be due to inhibition of renal tubular cation secretion rather than a true change in glomerular filtration rate [10]. However, dronedarone therapy has been associated with an increased risk of acute renal failure based on published case reports and adverse drug-reaction databases [11, 12]. The mechanism of renal failure is unknown but may be mediated by its multichannel blocking activity [11]. Serum creatinine may increase by more than 10 % during the first week of dronedarone therapy, an observation that does not necessarily herald impending renal failure. Nevertheless, progressive deterioration in kidney function based on laboratory measurements or clinical symptoms warrants discontinuation of dronedarone therapy and careful reassessment of renal function [13].

Dronedarone should be avoided or used cautiously with underlying permanent AF. Its use in this particular subgroup was associated with increased heart failure, stroke, and death from cardiovascular causes in a randomized trial of 3236 subjects [14]. The specific mechanism of this adverse effect remains unclear.

Dronedarone-associated hepatotoxicity was suggested in as many as 12 % of study patients who had an elevation in liver enzymes [2, 15]. Since market release, three cases of severe hepatitis have been reported with two requiring urgent liver transplantation [16, 17]. These cases prompted the FDA to issue a warning about possible severe hepatotoxicity with dronedarone use. No dosage adjustment is required in patients with mild-to-moderate hepatic impairment; however, dronedarone therapy is contraindicated in patients with severe hepatic dysfunction [13]. The mechanism of hepatic injury is likely similar to that of amiodarone and relates to drug-mediated inhibition of mitochondrial beta-oxidation and uncoupling of oxidative phosphorylation leading the cellular injury [17, 18].

## Conclusion

Novel therapies for drug-refractory ventricular arrhythmias are long overdue given the limitations of available pharmacologic and non-pharmacologic options. Off-label use of antiarrhythmic agents such as dronedarone is considered a treatment of last-resort in patients who otherwise have no therapeutic options. Given the paucity of reported cases regarding dronedarone for the treatment of ventricular tachyarrhythmias, no conclusive recommendations can be made at this time other than advising caution. Despite its potential ventricular antiarrhythmic effects careful patient evaluation is required to identify those at greatest risk of drug-related adverse events particularly in those patients with significant comorbidities such as advanced hepatic, renal, and cardiovascular disease.

## Abbreviations

AF: atrial fibrillation
LV: left ventricular
SCD: sudden cardiac death
VT: ventricular tachycardia
